# A Patient with Dyspnea and an Enlarged Right Ventricle

**DOI:** 10.14797/mdcvj.1124

**Published:** 2022-10-04

**Authors:** Michelle Nsahlai, Amr Telmesani, Valeria E. Duarte

**Affiliations:** 1 Houston Methodist Hospital, Houston, TX, US

**Keywords:** sinus venosus ASD, partial anomalous pulmonary venous return, left to right shunt

## Abstract

Case report of a patient with no significant past medical history who presented with reports of dizziness, dyspnea on exertion, and palpitations that had been ongoing for at least 5 years. It demonstrates the importance of considering the presence of an inter-atrial shunt when evaluating a patient with an unexplained dilated right atrium and right ventricle.

## Case presentation

A 38-year-old man with no significant past medical history presented to the emergency department with reports of dizziness, dyspnea on exertion, and palpitations that had been ongoing for at least 5 years. He reported that he felt dizzy several times a week, and his episodes of dizziness were unrelated to exertion. He said he had developed shortness of breath and palpitations in the past few months with activities such as brisk walking. He drank alcohol occasionally and denied smoking or using recreational drugs. He had no previous episodes of syncope and denied recent weight gain or viral infections. He reported that his father had a history of complete heart block and his mother had paroxysmal atrial fibrillation. He did not have a family history of sudden cardiac death, early-onset coronary artery disease, heart failure, or valvular heart disease.

His physical examination on admission revealed the following:

- Vitals: Temperature 96.8°F, pulse 64 bpm, blood pressure 125/78 mm Hg, respirations 17/min, oxygen saturation 97% on room air
- General: Well-nourished male in no acute distress
- HEENT: Normocephalic, atraumatic; no carotid bruits or jugular vein distention, no lymphadenopathy
- Heart: Regular rate and rhythm, soft low pitch mid-systolic murmurs at the upper left sternal border; no gallops
- Lungs: Normal work of breathing, clear to auscultation bilaterally
- Abdomen: Soft, nontender, and nondistended, with normal bowel sounds in all four quadrants; no hepatomegaly or splenomegaly
- Extremities: Trace bilateral lower extremity edema, 2+ pulses present throughout, no clubbing or cyanosis
- Skin: Warm and dry with no rashes
- Neurology: No focal abnormalities

**Figure 1:**
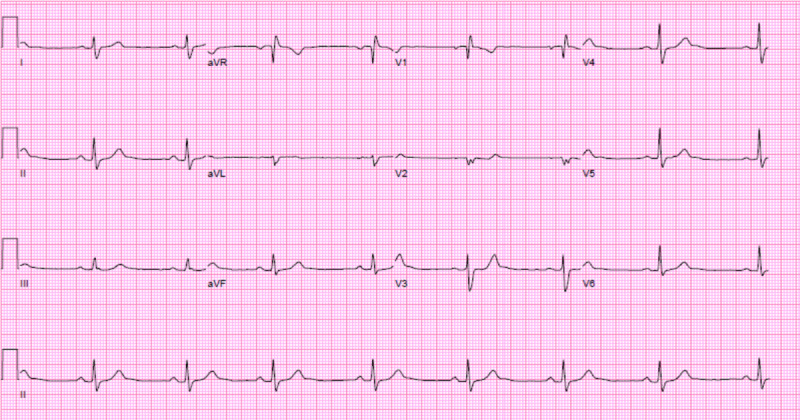
Electrocardiogram on presentation shows normal sinus rhythm, regular rate, and an incomplete right bundle branch block.

See Figure 1 for initial electrocardiogram. His initial laboratory findings were as follows:

- Complete blood count: White blood cells 10.7 k/uL, hemoglobin 14.6 g/dL, hematocrit 44.4 (MCV 87.7), platelets 252 K/uL
- Electrolytes: Sodium 142 mEq/L, potassium 3.9 mEq/L, magnesium 1.9 mEq/L, glucose 100 mg/dL, blood urea nitrogen 13 mg/dL, creatinine 0.87 mg/dL (at baseline)
- Normal liver function tests
- Troponin < 0.006 ng/L
- BNP 5 pg/mL
- HbA1c 5.5
- Urine toxicology was negative

**Figure 2:**
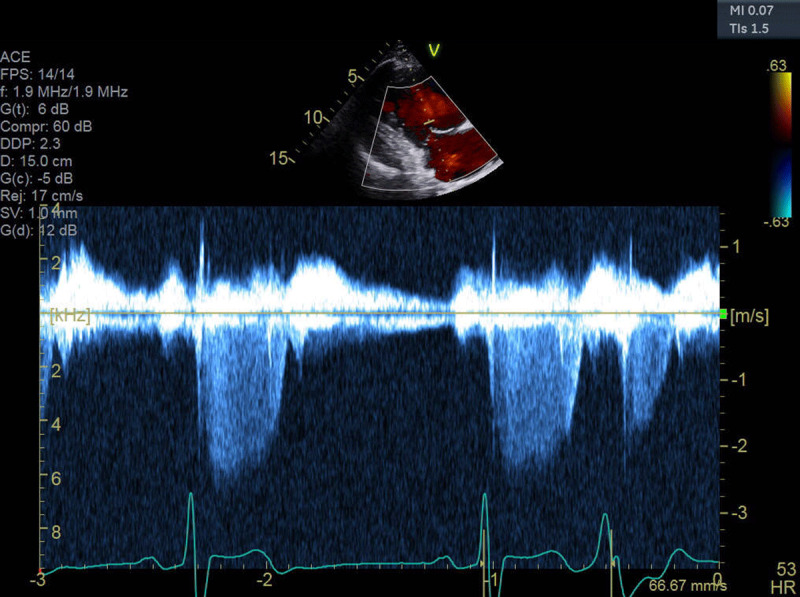
Tricuspid regurgitation velocity by continuous wave Doppler.

**Figure 3:**
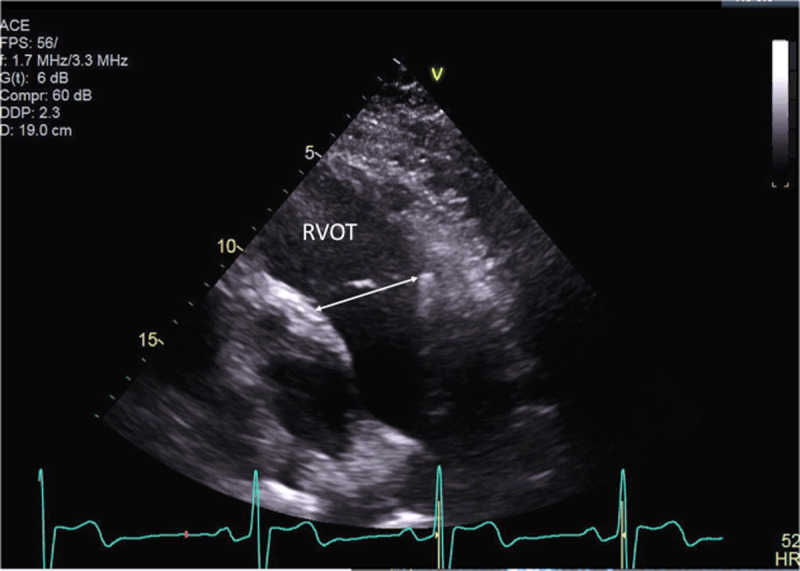
Right ventricular outflow tract dimension (3.8 cm).

**Figure 4:**
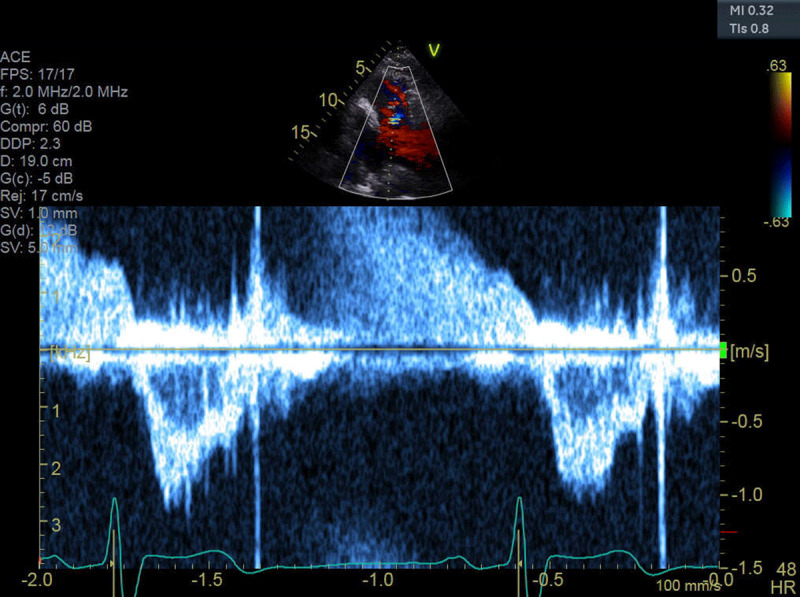
Right ventricular outflow velocity: TVI = 18 cm.

**Figure 5:**
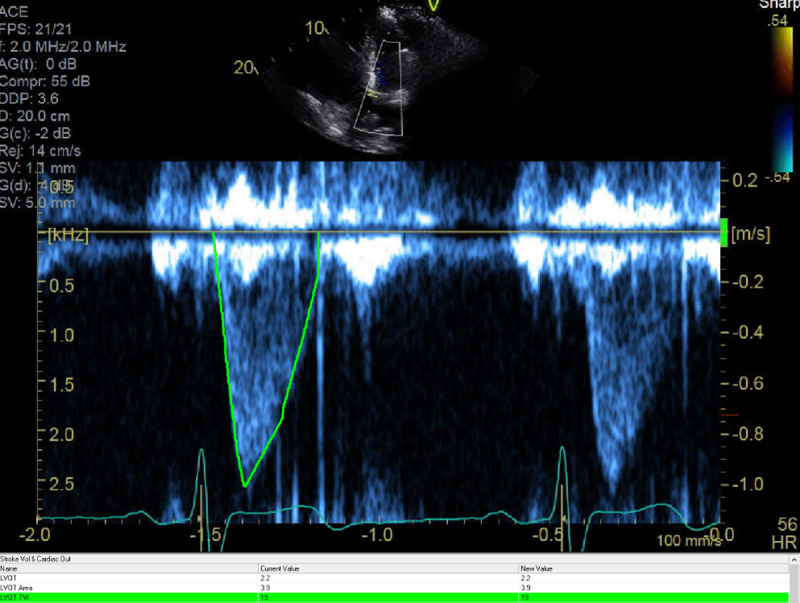
Left ventricular outflow velocity (TVI = 19 cm). The LV outflow diameter was 2.1 cm

The patient was not found to be orthostatic and was placed on a 30-day event monitor, which did not reveal any episodes of arrythmias. His symptoms persisted, so a transthoracic echocardiogram (TTE) was ordered (Videos 1, 2, 3, Figures 2, 3, 4, 5).

**Video 1:** Parasternal long-axis view, also at https://youtu.be/KEMSPVBHueM.

**Video 2:** Apical 4-chamber view with color Doppler, also at https://youtu.be/cypEgrn5Ut0.

**Video 3:** Subcostal view of the right and left atria, also at https://youtu.be/saQGbf_37Ts.

## Question 1

**The TTE showed:** 

- Normal findings and mild tricuspid regurgitation (TR)
- Normal chamber sizes, mild TR and right ventricular (RV) dysfunction
- Normal left ventricular (LV) function, mild TR, enlarged right ventricle, secundum atrial septal defect (ASD)
- Normal LV, enlarged RV, mild RV dysfunction, mild TR, no ASD seen.
- None of the above

**Question 1:** Consider the options and find the answer in this video quiz, also at https://youtu.be/EqjQCqCdgZI.

## Question 2

**What is the differential diagnosis of a dilated RV with reduced function?**

- Pulmonary arterial hypertension
- Arrhythmogenic right ventricular dysplasia
- RV infarction
- Interatrial shunt
- All of the above

**Question 2:** Consider the options and find the answer in this video quiz, also at https://youtu.be/AWA5TOtPqpw.

TR peak velocity was 2.5 m/s, indicating a pulmonary artery systolic pressure of 25 mm Hg plus the RA pressure, which was estimated at 5 mm Hg. With this finding, pulmonary hypertension is excluded as a cause for the RV enlargement.

## Question 3

**What is the most likely diagnosis?**

- Sinus venosus defect with a large left-to-right shunt
- RV infarction
- Secundum ASD with large left to right shunt
- Arrhythmogenic right ventricular dysplasia
- Severe TR missed by the color Doppler

**Question 3:** Consider the options and find the answer in this video quiz, also at https://youtu.be/TL9c2bfAq94.

**Figure 6:**
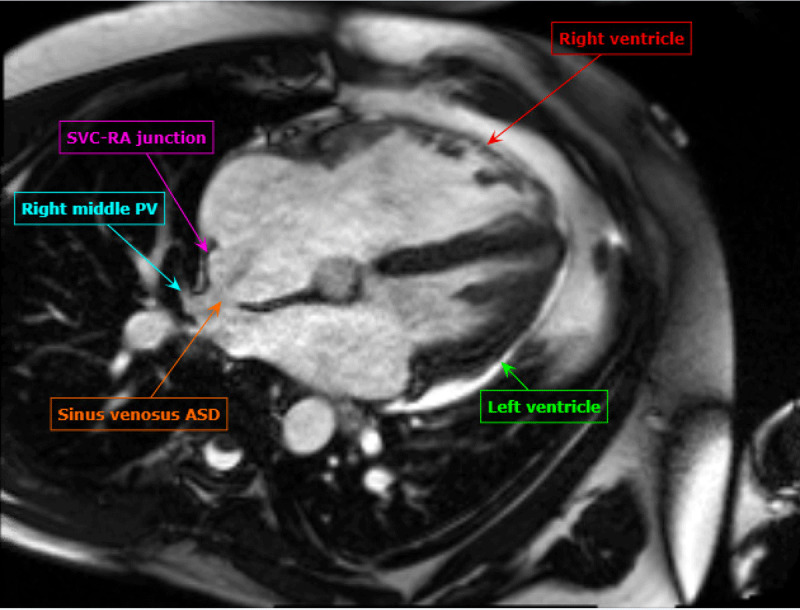
Four-chamber stack of cardiac magnetic resonance imaging showing sinus venosus atrial septal defect as labeled.

A CMR was performed (Figure 6) that showed a large superior sinus venosus defect (SVD) with partial anomalous venous return (PAPVR) of the right superior, right middle, and a branch of the right inferior pulmonary vein to the superior vena cava. There was a significant left-to-right shunt with a Qp:Qs of 2.0. The right ventricle was severely enlarged (end diastolic volume of 503 mL). There were no other associated congenital abnormalities noted.

The patient was referred to cardiothoracic surgery and underwent successful repair of the sinus venosus defect and PAPVR using the autologous pericardium to patch technique. This technique involves the creation of an intra-atrial baffle that is sewn to the SVC and used to redirect the anomalous pulmonary veins via the sinus venosus defect to the left atrium. A second piece of autologous pericardium is then used for closure of the superior vena cava and right atrial junction. The patient had no procedural complications and made a good recovery.

## Discussion

This case demonstrates the importance of considering the presence of an interatrial shunt when evaluating a patient with an unexplained dilated right atrium and right ventricle. Initial differential diagnosis included idiopathic pulmonary hypertension, right ventricular infarction, arrhythmogenic right ventricular dysplasia. and a large left-to-right shunt at atrial level. Physical examination showed fixed splitting of S2, and the ECG demonstrated an incomplete right bundle branch block, which are common findings with ASDs. The ECG did not reveal any evidence of RV hypertrophy, right axis deviation, and right atrial enlargement that would be expected in a patient with a longstanding history of pulmonary hypertension. There was no evidence of an old infarct in the inferior leads. The classic ECG findings for arrhythmogenic right ventricular dysplasia, which include T-wave inversions in the right precordial leads (V1-V3) with an epsilon wave after the QRS in lead V1, were also missing. TTE demonstrated a dilated RV with mild dysfunction and failed to show a secundum ASD. The most important finding came from the calculations of flows through the RV and LV outflow, which demonstrated a large left-to-right shunt. Without such calculations, the suspicion of a sinus venosus defect could have been missed. Sinus venosus defects are very challenging to diagnose with TTE; only one in four cases are correctly diagnosed.

Sinus venosus defect is a rare congenital heart disorder that occurs when there is communication between one or more of the pulmonary veins and the cardiac entrance of the SVC and/or the posterior-inferior wall of the right atrium.^6^ SVDs account for approximately 4% to 11% of ASDs and are almost always associated with anomalous pulmonary veins.^2,5,6^ From an anatomic perspective, SVD is not a true ASD because it does not allow direct communication between the left and right atria; rather, the shunt in SVD is through one or more systemic and pulmonary veins.^5^ The most common location of SVD is between the right upper pulmonary vein and the superior vena cava below the insertion of the Azygos vein (known as an SVC-type SVD), and it accounts for 87% of SVDs. Other types of SVDs include RA-type SVD, where the defect occurs at the junction of the inferior vena cava and the RA.^6^

Patients with SVDs often present with reports of dyspnea, fatigue, exercise intolerance, or palpitations.^2^ They can also present with syncope due to transient cerebral hypoperfusion, although that is less common. Approximately 20% of patients with ASDs have atrial arrhythmias, and the frequency increases as the patients age.^4^ Atrial arrhythmias can occur both before and after repair of the defect.^3^ Patients may remain largely asymptomatic for the first three decades and then develop symptoms around the fourth decade due to RV overload from left-to-right shunting.

The definite management of SVD with PAPVR is surgical repair, and it is indicated if the patient has impaired functional capacity, RA and/or RV enlargement, and the presence of a left-to-right shunt that is sufficient to cause physiological sequelae (Qp:Qs ≥ 1.5:1), provided the patient has not yet developed significant pulmonary hypertension.^1^ Cardiac catheterization is not required in every patient prior to surgical repair of an SVD, provided noninvasive imaging is of sufficiently high quality to estimate pulmonary artery pressures and shunt magnitude.^1,7^ Common risks associated with surgical repair of SVDs with PAPVR include sinus node dysfunction, SVC, and pulmonary vein obstruction. Nonetheless, early repair of SVASD and PAPVR improves morbidity and is generally associated with favorable outcomes.

## Key Points

- Perform a thorough clinical investigation when you see an unexplained large right atrium and right ventricle to ensure that a congenital atrial septal defect is not missed. This includes calculating flows through the right ventricular and left ventricular outflow tract to exclude a significant left-to-right shunt.
- If one congenital heart defect is identified, do a careful assessment to ensure that no other ones are present. Cardiac magnetic resonance (CMR) imaging is an excellent tool to accomplish this.
- Sinus venosus defects are not commonly diagnosed with transthoracic echocardiogram. When its presence is suspected, CMR or transesophageal echocardiogram are appropriate imaging modalities to confirm.
- Atrial arrhythmias are very common in patients with atrial septal defects both before and after repair.

## CME Credit Opportunity

Houston Methodist is accredited by the Accreditation Council for Continuing Medical Education (ACCME) to provide continuing medical education for physicians.

Houston Methodist designates this enduring material for a maximum of 1 AMA PRA Category 1 Credit™. Physicians should claim only the credit commensurate with the extent of their participation in the activity.

Click to earn CME credit: learn.houstonmethodist.org/MDCVJ-18.1.
